# Older age and a non‐sporting injury mechanism are associated with re‐injury and the need for revision surgery over a minimum 2‐year follow‐up following proximal hamstring tendon repair

**DOI:** 10.1002/ksa.12767

**Published:** 2025-07-07

**Authors:** Jay R. Ebert, Peter K. Edwards, Elias Ammann, Adam Farrier, Lorcan Gavin, Method Kabelitz, Ross Radic, Antony Liddell, Peter Annear

**Affiliations:** ^1^ School of Human Sciences (Exercise and Sport Science) University of Western Australia Perth Western Australia Australia; ^2^ HFRC Rehabilitation Clinic Perth Western Australia Australia; ^3^ Perth Orthopaedic & Sports Medicine Research Institute Perth Western Australia Australia; ^4^ School of Allied Health Curtin University Perth Western Australia Australia; ^5^ Perth Orthopaedic & Sports Medicine Centre Perth Western Australia Australia; ^6^ Department of Orthopaedic Surgery and Traumatology Kantonsspital Baselland Bruderholz Switzerland; ^7^ Royal Perth Hospital Perth Western Australia Australia; ^8^ Hollywood Private Hospital Perth Western Australia Australia; ^9^ Department of Orthopaedics Stadtspital Zürich Zürich Switzerland; ^10^ School of Medicine and Surgery University of Western Australia Perth Western Australia Australia

**Keywords:** predictive variables, proximal hamstring avulsion, revision surgery, surgical repair

## Abstract

**Purpose:**

To investigate revision rates and factors associated with the need for revision following proximal hamstring tendon repair.

**Methods:**

This study included 243 patients who underwent proximal hamstring tendon repair due to an acute (*n* = 176) or chronic (*n* = 67) tear. Complications, re‐injuries and re‐operations were reviewed. Risk factor analysis for re‐rupture within 2 years of surgery was conducted using Cox proportional hazards regression, with variables including age, body mass index (BMI), sex, mechanism of injury (sport‐related or other), time from injury to surgery and comorbidities including hypertension, hypercholesterolaemia and Type 2 diabetes. Receiver operating characteristic analysis explored time‐to‐surgery thresholds in relation to revision.

**Results:**

Overall, 19 (10.8%) of the acute cohort and 11 (16.4%) of the chronic cohort underwent revision surgery due to re‐tearing and recurrence of symptoms. In the acute cohort, an increased risk of re‐injury was associated with a non‐sporting (versus sporting) injury (hazard ratio [HR] = 3.38; 95% confidence interval [CI], 1.10–10.39; *p* = 0.033) and an older age (HR = 1.04 per year; 95% CI, 1.00–1.08; *p* = 0.031). In the chronic cohort, there were no significant associations between age, BMI, sex or comorbidities, with revision surgery. The optimal threshold for surgery for acute repairs was 30.5 days.

**Conclusions:**

A 10.8% and 16.4% revision rate was observed over a minimum 2‐year follow‐up following proximal hamstring repair for acute and chronic tears, respectively. For chronic tears, no variables were associated with the need for revision. However, older age and non‐sporting injury were associated with a higher risk of re‐injury in the acute cohort.

**Level of Evidence:**

Level IV, retrospective case series.

**Abbreviations**
BMI: body mass index
MRI: magnetic resonance imaging
RTS: return to sport

## INTRODUCTION

Hamstring injuries are common in both athletic [7] and general [13] populations, often due to a stretch‐type injury caused by extensive hip flexion with an extended knee, or with running during the eccentric contraction required in the late swing phase of gait [18]. It is reported that 12% of these result in a rupture of the proximal tendon attachment [12]. Systematic reviews have shown that surgical repair of these ruptures results in improved clinical scores, high patient satisfaction, good muscle strength restoration and ability to return to sport (RTS) [3, 5, 6, 8, 9, 20]. However, repair outcomes for chronic (vs. acute) ruptures are often less favourable [3, 8, 9]. Non‐surgical management may often be attempted in a range of proximal hamstring pathologies [1] and indeed in chronic cases. However, many patients may eventually proceed towards surgery due to persistent pain, reduced strength and limited sports performance [8].

After proximal hamstring tendon repair, re‐rupture rates across different studies are varied, though pooled re‐rupture rates in systematic reviews spanning the repair of acute and chronic tears have been reported at 1.2% [9], 2.2% [3] and 2.7% [8], which are generally higher for chronic tears [9]. One of the challenges is the lack of a clear definition of tear chronicity. While a recent study reported that a longer initial surgery delay was a risk factor for re‐injury after proximal hamstring tendon repair [16], with an optimal injury‐surgery cut‐off >32 days as predictive of re‐rupture, extensive variation has been reported in the literature. Across varied studies, tear chronicity has been defined as a surgical delay >4 weeks [11, 15, 21], >6 weeks [2, 4], >8 weeks [20] and >3 months [22].

Bowman et al. [4] reported that clinical outcomes remain variable, and there is limited data on which patients are most likely to have a favourable outcome after repair. They investigated the role of various factors, including patient demographics, medical comorbidities, tear characteristics and repair technique, on clinical scores, satisfaction and timing of RTS after proximal hamstring repair, with no differences in outcomes found based on these variables, with the patient numbers available. A study investigating the association between patient‐related factors and the risk of re‐rupture and/or revision surgery after primary repair is yet to be undertaken. This information would provide a means of accurate preoperative patient education and setting of realistic expectations and risk of revision surgery.

This study investigated revision rates and the contribution of pertinent patient demographics and medical comorbidities to the need for revision surgery due to persistent or recurrent symptoms, within at least 2 years of undergoing primary repair for acute or chronic proximal hamstring tears. Furthermore, it sought to explore an optimal threshold for the injury‐surgery timeframe with respect to re‐injury and the need for revision surgery. The primary hypothesis was that in both the acute and chronic repair settings, factors that are associated with the need for revision surgery would be identified.

## MATERIALS AND METHODS

### Patients

**Figure 1:**
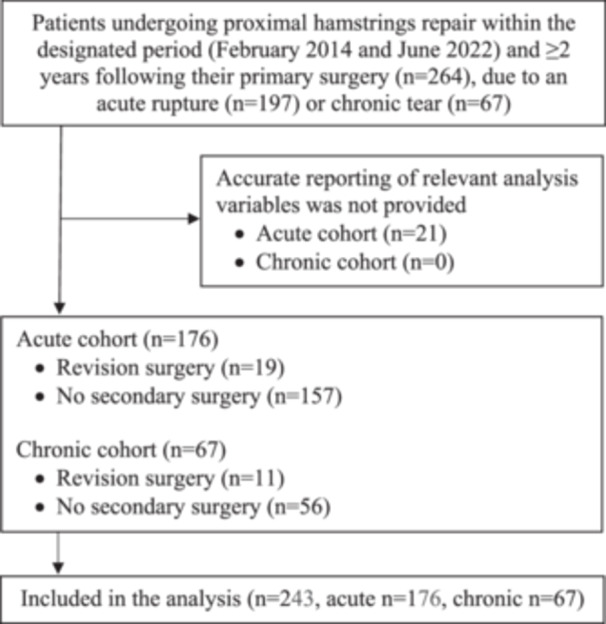
Flowchart demonstrating patients undergoing proximal hamstrings repair due to an acute rupture or chronic tear within the designated period and included in the current analysis.

This retrospective study included 243 patients who underwent open primary proximal hamstring tendon repair surgery by one of three orthopaedic surgeons (operating within two private hospitals), between February 2014 and June 2022, due to an acute rupture (*n* = 176) or chronic tear (*n* = 67) (Figure 1). Patients were followed up within a mean of 5.4 years (range: 2.0–11.4) of their primary repair. The retrospective study analysis was approved by the institutional Human Research Ethics Committee (HREC).

Initially, all patients who had undergone proximal hamstring repair from the three orthopaedic surgeons involved over the designated period were identified via surgical and institutional databases. Patient demographics (sex, age, height, weight, body mass index [BMI]), injury history (injury mechanism and time from injury to surgery specifically for acute repairs), and medical comorbidities (hypertension, Type 2 diabetes and hypercholesterolaemia) were obtained from institutional and hospital medical records.

While all patients who underwent primary proximal hamstring repair surgery within the designated period were included in the analysis, surgical inclusion criteria included skeletally mature patients presenting to the orthopaedic surgeon with symptoms associated with proximal hamstring rupture, with the diagnosis confirmed in all patients via magnetic resonance imaging (MRI). As mentioned above, and despite the reported variation in the definition of an ‘acute’ injury, acute repairs in the current analysis were generally defined as <8 weeks from injury, though all of these were from a specific traumatic event.

### Surgical procedures

Briefly, all surgical repairs were undertaken with the patient positioned in the prone position, under a general anaesthetic. Routine preparation and draping were undertaken, with the leg free‐draped to allow exposure of the proximal hamstring.

An oblique incision was made in line with the gluteal fold, or alternatively a longitudinal incision was employed depending on surgeons' preference. In instances of severe retraction of the tendon or chronic cases, longitudinal incisions may have been employed to allow distal exposure of the retracted tendon and mobilization.

Direct dissection through subcutaneous layers was undertaken, preserving the posterior cutaneous branch of the thigh. A Hohmann retractor was placed underneath the gluteal fold to elevate the gluteus maximus and allow exposure of the ischial tuberosity and hamstring origin. The deep fascia overlying the hamstring tendon was incised, exposing free blood‐stained fluid in acute cases. The sciatic nerve, lying lateral to the ischial tuberosity and hamstring tendons, was identified at this stage, with neurolysis performed when required and the nerve protected through surgery.

The avulsed tendon was then identified and mobilized to allow approximation to the hamstring footprint on the posterior and lateral aspect of the ischial tuberosity. The ischium was then freshened to bleeding bone to enhance tendon‐bone healing, and a double‐row anchor construct was utilized to repair the avulsed tendon to its native footprint. Two 5.5‐mm double‐loaded corkscrew anchors (Arthrex) were used in the deep (anterior) attachment, with the firewire sutures passed through two 4.75/5.5 mm swivel locks (Arthrex) at the superficial (posterior) portion of the hamstring attachment. Stable fixation of the attachment was then confirmed, while sciatic nerve integrity was confirmed with direct observation. The incision was closed in layers with 1 vicryl, 2‐0 vicryl and 3‐0 monocryl running sutures. Finally, 10 mL of 0.75% naropin was infiltrated into the wound with routine dressings applied.

### Post‐operative management

Post‐operatively, patients were instructed to partially weight bear with crutches for the first two weeks to allow pain relief, comfort and wound healing. At 2 weeks post‐surgery, patients were generally permitted to wean from crutches, with unaided ambulation routinely achieved 2–4 weeks post‐operatively. This early 4‐week period was standardized across all three surgeons, consulting out of the same private practice. Therapist‐led rehabilitation was generally initiated from this time, with an initial focus on the initiation of passive (and active) hip and knee range of motion (ROM) exercises, isometric quadriceps and gluteal exercises and gait retraining. An individualized progression through varied open and closed kinetic chain exercises was undertaken, with guided progression through plyometric and jump‐based activities if relevant. A return to running was permitted no earlier than 6–8 weeks post‐operatively, while a return to unrestricted activities was permitted no earlier than 3 months. Of note, the progression of strength and conditioning activities, along with the graduated re‐introduction of various recreational and/or sporting activities, was undertaken under the ongoing direction of the patient's own outpatient therapist. While each of the three surgeons had specific physical therapists to whom they referred patients undergoing proximal hamstring repair, these physical therapists were not consistent across the three surgeons involved.

### Statistical analysis

First, complications were collected from all patients, and subsequently categorized and reported as major or minor complications [14]. As previously reported, minor complications were those that caused persistent symptoms without significant impairment (such as superficial wound infection, transient nerve injury, haematoma and peri‐incisional numbness), while major complications were those that caused debilitating injury, required further surgery, or were potentially life threatening (such as re‐rupture, sciatic nerve injury, deep vein thrombosis and deep infection). Revision rates following primary repair with at least 2 years of follow‐up, due to persistent or recurrent symptoms, were presented for the acute and chronic cohorts. Descriptive statistics were reported as frequencies and percentages for categorical variables. Continuous variables were presented as means and standard deviations for normally distributed data, or medians and interquartile ranges for non‐normally distributed data. Risk factor analysis was performed using Cox proportional hazards regression. Variables with a univariate association with revision surgery at *p* < 0.10 were considered for inclusion in the multivariate model. To explore potential time‐to‐surgery thresholds associated with revision, receiver operating characteristic (ROC) analysis and the Youden index were used to identify optimal cut‐points. Kaplan–Meier survival curves were generated to compare revision‐free survival between groups, with differences assessed using the log‐rank test. Time was measured in months. Survival analyses were also undertaken for specific 14‐ and 32‐day time‐to‐surgery cut‐offs, as employed in previous published work [16]. The identified variables from the univariate Cox regression analyses were included in the multivariate Cox regression model. A backwards stepwise method was used to eliminate non‐significant variables from the model, with a threshold of *p* < 0.05 used for retention in the model. The proportional hazards assumption was checked using time‐dependent covariates, and no violations were identified. Multicollinearity was assessed using variance inflation factors, with no evidence of collinearity observed. All analyses were conducted using SPSS (IBM SPSS Statistics, Version 31.0), with a two‐tailed significance threshold of *p* < 0.05.

## RESULTS

Of the 264 patients who underwent surgery over the designated period, 243 (176 acute, 67 chronic) were included in the current analysis (Figure 1). Those that were omitted were all in the acute group for reasons of incomplete baseline data collection (demographics and/or injury date). Characteristics of the included cohort are presented in Table 1. The reported mechanism of injury in the acute group included during sports or sport‐related activities (*n* = 91), during slip or fall during activities of daily living (*n* = 59), following a higher energy incident, including motor vehicle accidents (*n* = 8) and other (*n* = 18).

**Table 1:** Patient demographics for the cohort that underwent proximal hamstring tendon repair due to an acute (*n* = 176) or chronic (*n* = 67) injury.

	Acute repair cohort (*n* = 176)				Chronic repair cohort (*n* = 67)			
	No revision	Revision	Hazard ratio (95% CI)	*p*	No revision	Revision	Hazard ratio (95% CI)	*p* value
*n*	157 (89.2)	19 (10.8)	N/A	N/A	56 (83.6)	11 (16.4)	N/A	N/A
Age (years)	48.8 ± 15.0	53.2 ± 16.3	1.04 (1.00–1.08)	0.031	61.2 ± 9.6	56.3 ± 7.6	0.96 (0.90–1.02)	0.190
Body mass index	26.5 ± 4.5	26.2 ± 5.4	1.03 (0.94–1.14)	0.512	26.3 ± 4.4	24.1 ± 4.1	0.94 (0.81–1.09)	0.439
Sex (male)	93 (59.2)	8 (42.1)	0.45 (0.18–1.17)	0.102	11 (19.6)	2 (18.2)	0.94 (0.20–4.43)	0.938
Time to surgery, (days), median (IQR)	26 (14–52)	41 (15–56)	1.00 (0.99–1.01)	0.614	N/A	N/A	N/A	N/A
Mechanism								
Sport‐related	85 (54.1%)	6 (31.6%)	Reference		N/A	N/A	N/A	N/A
Non‐sport related	72 (45.9%)	13 (68.4%)	3.38 (1.10–10.39)	0.033	N/A	N/A	N/A	N/A
Hypothyroidism	7 (4.5)	1 (5.3)	1.46 (0.19–10.89)	0.716	0 (0.0)	0 (0.0)	N/A	N/A
Hypertension	21 (13.4)	4 (21.1)	2.00 (0.65–6.1)	0.227	15 (26.8)	3 (27.3)	1.00 (0.25–3.91)	0.997
Hypercholesterolaemia	12 (7.6)	1 (5.3)	0.82 (0.11–6.19)	0.848	8 (14.3)	0 (0.0)	N/A	N/A
Type 2 diabetes mellitus	1 (0.6)	1 (5.3)	8.33 (1.10–63.17)	0.04	5 (8.9)	0 (0.0)	N/A	N/A

Abbreviations: CI, confidence interval; IQR, interquartile range.

### Acute repair cohort—Complications, re‐injuries and revision surgeries

Of the 176 acute repair patients included, minor complications included superficial wound infection (*n* = 8, all of which responded to oral antibiotics), peri‐incisional numbness (*n* = 8), haematoma (*n* = 1) and transient nerve injury (*n* = 2). Three patients experienced deep vein thrombosis. One patient underwent surgical wound washout at 5 days post‐surgery, while one underwent surgical evacuation of a haematoma, with both patients having no ongoing issues. A total of 19 patients (10.8%) underwent revision surgery due to persistent pain and symptoms associated with re‐rupture, with revision procedures undertaken at a mean of 23.1 months (range 3 months to 6.6 years) after primary repair. Re‐injuries (and the need for re‐operation) were due to another new sporting incident (n = 6) or a different traumatic incident, such as a slip or fall (*n* = 6), with the remaining patients (n = 7) reporting persistent and increasing post‐surgery symptoms that refused to settle or a recurrence of symptoms without a definitive cause.

### Chronic repair cohort—Complications, re‐injuries and revision surgeries

Of the 67 chronic repair patients, minor complications included superficial wound infection (*n* = 3, all of which responded to oral antibiotics) and peri‐incisional numbness (*n* = 2). Furthermore, a total of 11 patients (16.4%) underwent revision surgery due to persistent pain and symptoms associated with re‐rupture, with revision procedures undertaken at a mean of 22.4 months (range 6 months to 9.7 years) after primary repair. Of these, a small cohort (*n* = 4) experienced a new traumatic incident (such as a slip or fall), though the remaining patients (*n* = 7) either reported ongoing symptoms post‐surgery that continued to escalate or had a recurrence of symptoms without a definitive cause.

### Univariate analysis of potential risk factors

In the acute repair cohort (*n* = 176), none of the assessed baseline variables, such as BMI, sex, hypertension, hypercholesterolaemia or hypothyroidism, were significantly associated (*p* > 0.05) with the risk of revision surgery in univariate Cox regression analysis (Table 1). However, patients who experienced a primary acute tear as a result of a non‐sporting injury (such as from a fall or during normal ADLs) had a significantly higher risk of revision compared to those injured during sport (hazard ratio [HR] = 3.38; 95% confidence interval [CI], 1.10–10.39; *p* = 0.033) (Table 1). Older age was also associated with increased risk (HR = 1.04 per year; 95% CI, 1.00–1.08; *p* = 0.031) (Table 1). Time from injury to surgery was not associated with revision risk in this cohort (HR = 1.00; 95% CI, 0.99–1.01; *p* = 0.614) (Table 1). In the chronic repair cohort (*n* = 67), there were no significant associations between age, BMI, sex or comorbidities and revision surgery (Table 1). All univariate hazard ratios had wide confidence intervals and *p* > 0.10, and no variables met criteria for inclusion in a multivariate model. Due to the low number of revisions (*n* = 11) and the absence of significant associations in univariate Cox regression, a multivariable model was not performed for the chronic repair cohort.

### Time to surgery and the effect on risk of revision surgery

**Figure 2:**
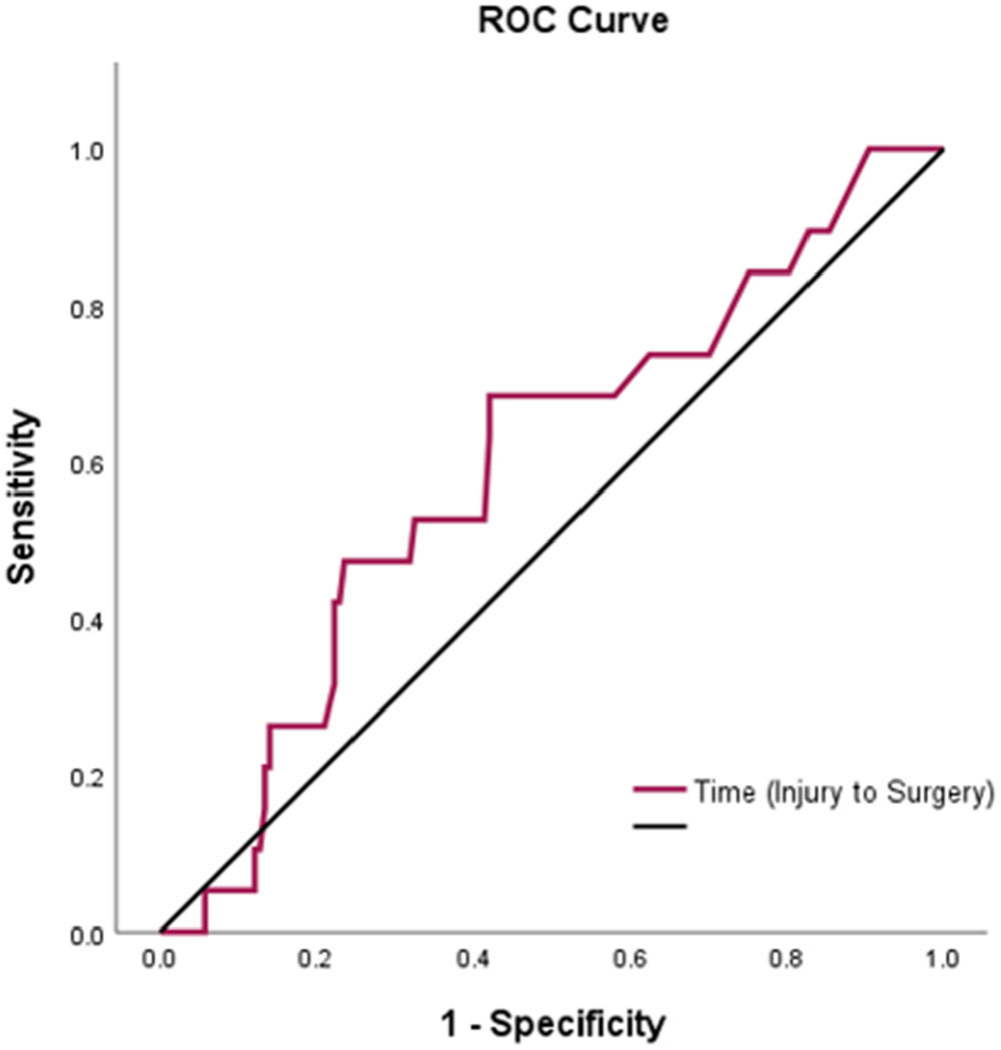
Receiver operating characteristic (ROC) analysis to evaluate the ability of time from injury to surgery to discriminate between patients who did and did not experience re‐injury.

ROC analysis was conducted to evaluate whether the time from injury to surgery could discriminate between patients who did and did not experience re‐injury. The area under the curve (AUC) was 0.60 (95% CI, 0.46–0.73; *p* = 0.155) (Figure 2), indicating poor discrimination. The optimal threshold, identified using the Youden index, was 30.5 days, corresponding to a sensitivity of 68.4%, a specificity of 58.0% and a Youden index of 0.264. Although the AUC was not statistically significant, the Youden index was applied to explore a potential threshold for clinical interpretation.

**Figure 3:**
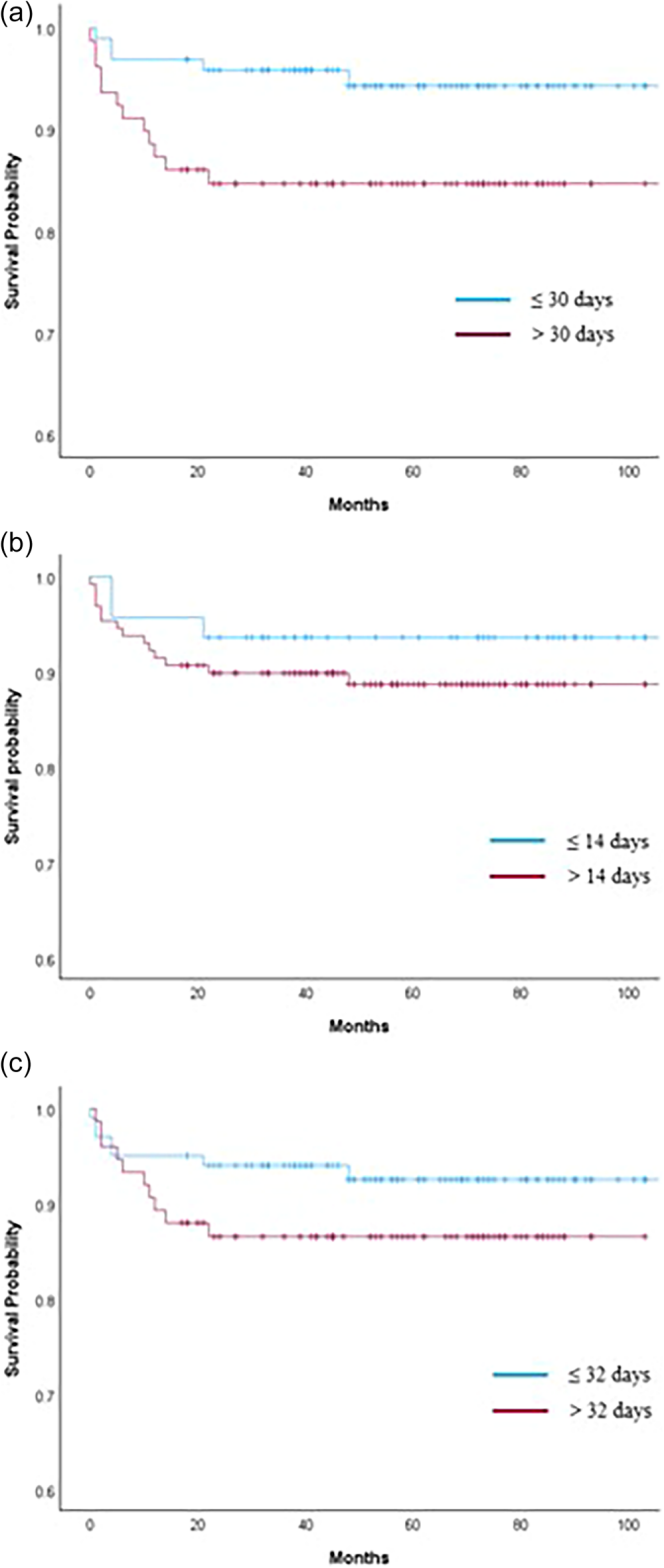
Kaplan–Meier survival analyses showing survival (re‐injury/re‐operation) based on time from injury to surgery, comparing: (a) ≤30 days versus >30 days, (b) ≤14 days versus >14 days and (c) ≤32 days versus >32 days.

Among the tested cut‐off points, Cox regression analysis showed that patients who underwent surgery beyond 14, 30 and 32 days, did not experience a statistically significant increase in revision risk. The HRs for surgery beyond each cut‐off were 1.41 (95% CI, 0.46–4.32; *p* = 0.547) for 14 days, 2.58 (95% CI, 0.97–6.88; *p* = 0.058) for 30 days and 1.97 (95% CI, 0.75–5.12; *p* = 0.168) for 32 days, respectively (Table 2). The Kaplan–Meier survival analysis comparing (≤30 days) versus delayed (>30 days) showed no statistically significant difference in revision‐free survival between the two groups (log‐rank test; *p* = 0.049) (Figure 3a). Specifically, the revision rate was 6.2% ≤30 days from time to surgery, and 12.5% >30 days. Survival analyses using 14‐ and 32‐day cut‐offs as per previous studies [16] showed no significant differences in revision‐free survival between early and delayed surgery groups (log‐rank *p* = 0.545 and *p* = 0.159, respectively), despite slightly higher revision rates in the delayed groups (Figure 3b,c).

**Table 2:** Cox regression modelling demonstrating revision risk associated with cut‐off time points points of 14, 30 and 32 days from injury to surgery.

Cut‐off definition	Revision/total	HR (95% CI)	*p* value
14‐day cut‐off			
Time to surgery ≤ 14 days	4/47	Reference	
Time to surgery > 14 days	15/129	1.41 (0.46–4.32)	0.547
30‐day cut‐off			
Time to surgery ≤ 30 days	6/97	Reference	
Time to surgery > 30 days	13/79	2.58 (0.97–6.88)	0.058
32‐day cut‐off			
Time to surgery ≤ 32 days	9/101	Reference	
Time to surgery > 32 days	10/75	1.97 (0.75–5.12)	0.168

### Final multivariate Cox regression analysis

In the univariate model, age, sex, injury mechanism and diabetes were identified as the potential confounders associated with the outcome (*p* ≤ 0.10) (Table 2). Additionally, a time to surgery cut‐off of >30 days was also identified as a potential factor for revision surgery and was also included in the final multivariate model. An injury‐to‐surgery delay of >30 days was associated with a 2.4‐fold increased risk of revision (HR = 2.40; 95% CI, 0.90–6.42; *p* = 0.08), while a non‐sporting mechanism was associated with a 3.44‐fold higher risk of revision compared with sport‐related injuries (HR = 3.44; 95% CI, 1.12–10.58; *p* = 0.031).

## DISCUSSION

The current study has demonstrated a 10.8% and 16.4% rate of revision surgery (due to persistent or recurrent symptoms with evidence of re‐tearing) over a minimum follow‐up of 2 years following primary proximal hamstring repair for acute and chronic tears, respectively. While no significant associations were observed between the risk or re‐injury and relevant demographics and health comorbidities in the chronic cohort, in the acute cohort an increased risk of revision surgery was associated with a non‐sporting (versus sporting) injury and an older age, though time from injury to surgery (at least within the designated 8‐week ‘acute’ timeframe) was not associated with revision risk. These findings will permit a more targeted discussion with patients undergoing surgery, with respect to the incidence of re‐injury and potential need for revision surgery, as well as how this may relate to them when considering their own demographics and injury history.

Encouraging clinical outcomes have been reported in patients undergoing operative repair for proximal hamstring tears [3, 5, 8, 9, 20], though outcomes in the chronic (vs. acute) setting have generally been less favourable [3, 8, 9]. A systematic review published by Harris et al. [8] in 2011 reported that repair in the acute (vs. chronic) setting resulted in significantly better patient satisfaction, clinical outcomes and return to pre‐injury sport rates, along with a lower risk of complication and/or re‐rupture. While a review published by van der Made et al. [20] in 2014 reported no to minimal difference in satisfaction, pain, strength and RTS in the acute or delayed surgical setting, more recent reviews by Bodendorfer et al. [3] in 2017 and Hillier‐Smith and Paton [9] in 2022 reported that repair of an acute (versus chronic) avulsion provided less pain and improved satisfaction, clinical scores and strength, as well as a significantly lower re‐rupture rate. While re‐rupture rates are varied across studies, pooled re‐rupture rates in systematic reviews spanning the repair of acute and chronic tears have been reported at 1.2% [9], 2.2% [3] and 2.7% [8], which are generally higher for chronic (vs. acute) tears [9]. These pooled re‐rupture rates are lower than the rate reported in the current study.

Despite the growing body of evidence reporting clinical, functional and RTS outcomes after proximal hamstring repair, there is limited data on which patients are most likely to have a favourable outcome after repair [4]. In the current study and specific to the cohort that underwent proximal hamstring repair in the context of an acute injury, 10.8% underwent revision surgery within at least 2 years of undergoing primary repair, which is certainly higher than what had been reported in the most recently published systematic review and meta‐analysis on the topic [9]. While the reasons for re‐injury and the need for revision surgery were documented in the current study, irrespective of the cohort (acute or chronic, with 30 revision surgeries overall) roughly half of these were either from a new sporting accident (*n* = 6, 32%) or a new traumatic slip or fall (*n* = 6, 32%), with the rest either having persistent and worsening symptoms following surgery or recurrence of symptoms without a definitive cause. Regardless, the reasons for the differences in revision rates between the current study and existing literature is not fully known, though may include differences between cohorts (e.g., the current study included three‐tendon injuries where many of the aforementioned studies include partial tears), the lengthier follow‐up in the current study (2–11 years in the current cohort), and possibly a lower threshold to undertake revision surgery by the surgeons involved in the current study. Furthermore, it should be acknowledged that the cohort omitted from the analysis due to the lack of required baseline data had not undergone revision surgery, so this will further overinflate the reported revision rates.

Of interest, Lefevre et al. [16] recently reported a 4.6% re‐rupture rate after proximal hamstring repair, with most of their re‐ruptures occurring within the first 6 months, with the current study reporting a mean re‐operation timeframe of 23.1 months (range 3 months to 6.6 years) in the acute cohort and 22.4 months (range 6 months to 9.7 years) in the chronic cohort. Further to this, in the acute cohort, an older age and non‐sporting (vs. sporting) injury were associated with a higher re‐injury risk, though time from injury to surgery (at least within the designated 8‐week ‘acute’ timeframe) was not associated with revision risk. Lefevre et al. [16] reported that a longer initial surgery delay was associated with an increased re‐rupture risk, and further reported an optimal injury‐surgery delay cut‐off predicting re‐rupture to be 32 days. Similarly, the current study observed a cut‐off of 30.5 days, though relatively poor discrimination, sensitivity and specificity were observed.

It has been reported that re‐rupture rates may be higher after surgery for chronic versus acute tears [9], though recent studies reporting outcomes specific to surgery for chronic proximal hamstring tendon avulsions have reported high functional outcomes and satisfaction at a minimum 2‐year follow‐up [10, 19], with no report of failure or symptom recurrence in these studies. Specific to the current study, no variables were investigated that were shown to be a significant predictor of revision proximal hamstring repair in the chronic tear setting. Li et al. [17] reported that increasing age was a significant predictor of poorer clinical outcomes after repair of chronic proximal hamstring tendon ruptures, though of interest, fatty atrophy of the hamstring musculature on preoperative MRI, assessed via the Goutallier classification system, was not associated with post‐operative clinical outcome. Bowman et al. [4] sought to evaluate the role of various factors, including patient demographics, medical comorbidities, tear characteristics and repair technique on clinical scores, satisfaction and timing of RTS after proximal hamstring repair. They were unable to detect any differences in outcomes based on these variables, although the cohort appeared to include both acute and chronic tear aetiologies, and the cohort only included 58 patients, of whom 45 had patient‐reported outcome measures at the final follow‐up point. Furthermore, they did not look at how these factors may be associated with re‐injury and/or the need for revision surgery.

Several study limitations are acknowledged in the current study. While the cohort includes the largest sample investigated and a study of this nature is yet to be undertaken, the retrospective study design should be acknowledged, which precludes the inclusion of other predictive variables that were not collected. Future prospective cohorts should seek to investigate the role of other patient‐related factors in post‐operative outcomes, satisfaction, repair failures and revision surgery, such as tendon quality at the time of surgery, and post‐operative recovery of strength and function. Second, all patients included in the current study underwent surgery via an open approach, the preferred method of the three surgeons involved. While a recent study reported complications in patients undergoing proximal hamstring tendon repair, with the majority of studies included via an open surgical approach (37 studies for open repair, 3 studies for endoscopic repair, and 3 studies combining open and endoscopic repair) [14], the results of the current study cannot be generalized across all surgical approaches. Third, following the early 4‐week post‐operative period, rehabilitation was not standardized. While each of the three surgeons had specific physical therapists to whom they referred patients undergoing proximal hamstring repair, these therapists were not consistent across the three surgeons, which introduces variation in each patient's individual progression. Fourthly, this study sought to investigate the role of certain variables on the need for revision surgery due to persistent/recurrent symptoms in the presence of MRI‐based failure, rather than surgical failure itself. It may well be that a cohort of the group that underwent surgery for acute or chronic tears may be asymptomatic in the presence of undiagnosed surgical failure.

As for clinical relevance, the current study reported a 10.8% and 16.4% revision rate (within a minimum 2‐year follow‐up period) in patients undergoing proximal hamstring repair for acute and chronic tears, respectively. While none of the variables employed were associated with the need for revision in patients undergoing repair for chronic tears, older age and non‐sporting injury were associated with a higher risk of re‐injury in patients undergoing repair for acute tears with a specific traumatic mechanism. Future studies should aim to employ a more robust array of variables, such as pre‐ and post‐operative activity and occupational history, as well as strength and functional capacity, quality and adherence to rehabilitation, and other specifics relevant to the surgery, such as tendon quality at the time of the procedure. Furthermore, future studies should be prospective in nature and also include serial MRI to evaluate the integrity of the repair, especially given that failure can likely occur without a significant recurrence of symptoms that may require revision surgery.

## CONCLUSION

A 10.8% and 16.4% revision rate was observed over a minimum 2‐year follow‐up following proximal hamstring repair for acute and chronic tears, respectively. For chronic tears, no variables were associated with the need for revision, though older age and non‐sporting injury were associated with a higher risk of re‐injury in the acute cohort.

## AUTHOR CONTRIBUTIONS

The following authors have conceived and designed the study (Jay R. Ebert, Elias Ammann, Antony Liddell and Peter Annear), supervised the conduct of the study (Jay R. Ebert, Peter K. Edwards, Elias Ammann, Adam Farrier, Lorcan Gavin, Antony Liddell and Peter Annear), analysed the data (Jay R. Ebert, Peter K. Edwards, Elias Ammann, Adam Farrier, Lorcan Gavin and Method Kabelitz), wrote the initial drafts (Jay R. Ebert, Peter K. Edwards and Elias Ammann), critically revised the manuscript (All authors) and ensure the accuracy of the data and analysis (All authors). I confirm that all authors have seen and agree with the contents of the manuscript and agree that the work has not been submitted or published elsewhere in whole or part.

## CONFLICT OF INTEREST STATEMENT

The authors declare no conflicts of interest.

## ETHICS STATEMENT

Ethics approval was obtained by the University of Western Australia (UWA) Human Research Ethics Committee (HREC (RA/4/20/4824)).

## Data Availability

Data have not been made publicly available, though data sets generated during the current study can be made available from the corresponding author on reasonable request.
